# Role of the median preoptic nucleus in the development of chronic deoxycorticosterone acetate (DOCA)‐salt hypertension

**DOI:** 10.14814/phy2.16046

**Published:** 2024-05-15

**Authors:** John P. Collister, Nora Fritcher, David Nahey

**Affiliations:** ^1^ Department of Veterinary and Biomedical Sciences, College of Veterinary Medicine University of Minnesota St. Paul Minnesota USA

**Keywords:** DOCA salt, hypertension, median preoptic nucleus, sympathetic nervous system

## Abstract

We have previously reported that the subfornical organ (SFO) does not contribute to the chronic hypertensive response to DOCA‐salt in rats, and yet the organum vasculosum of the lamina terminalis (OVLT) plays a significant role in the development of deoxycorticosterone acetate (DOCA)‐salt hypertension. Since efferent fibers of the OVLT project to and through the median preoptic nucleus (MnPO), the present study was designed to test the hypothesis that the MnPO is necessary for DOCA‐salt hypertension in the rat. Male Sprague‐Dawley rats underwent SHAM (MnPOsham; *n* = 5) or electrolytic lesion of the MnPO (MnPOx; *n* = 7) followed by subsequent unilateral nephrectomy and telemetry instrumentation. After recovery and during the experimental protocol, rats consumed a 0.1% NaCl diet and 0.9% NaCl drinking solution. Mean arterial pressure (MAP) was recorded telemetrically 5 days before and 21 days after DOCA implantation (100 mg/rat; SQ). The chronic pressor response to DOCA was attenuated in MnPOx rats by Day 11 of treatment and continued such that MAP increased 25 ± 3 mmHg in MnPOsham rats by Day 21 of DOCA compared to 14 ± 3 mmHg in MnPOx rats. These results support the hypothesis that the MnPO is an important brain site of action and necessary for the full development of DOCA‐salt hypertension in the rat.

## 1 INTRODUCTION

The DOCA‐salt hypertension model has been the subject of extensive research over the years, and a substantial body of evidence suggests a significant neurogenic component within this model. This component primarily involves the central nervous system and subsequent activation of elements within the sympathetic nervous system. To illustrate, sustained intracerebroventricular (ICV) administration of aldosterone induces hypertension (Gomez‐Sanchez, 1986). Moreover, mineralocorticoid receptors and epithelial sodium channels have been identified throughout the lamina terminalis of the brain (Amin et al., 2005; Miller et al., 2013), and blocking central mineralocorticoid receptors or epithelial sodium channels has been shown to prevent aldosterone or DOCA‐induced hypertension (Abrams et al., 2010; Gomez‐Sanchez et al., 1990; Nishimura et al., 1998). Additionally, concerning the role of downstream sympathetic nervous system activity in this model, both renal and splanchnic denervation have been found to mitigate DOCA‐induced hypertension in rats (Banek et al., 2016; Jacob et al., 2005; Kandlikar & Fink, 2011). Despite these findings, the specific central structures responsible for the hypertensive response to DOCA and the associated sympathetic activation remain unclear.

The anteroventral portion lining the third ventricle (AV3V) in the hypothalamus has long been recognized for its crucial role in maintaining homeostasis, regulating thirst, responding to osmotic changes, and controlling blood pressure. Notably, the AV3V has been demonstrated to be essential for the development of various forms of experimental hypertension (Brody, 1988), including the hypertensive reaction to DOCA administration in rats (Buggy et al., 1977; Songu‐Mize et al., 1982). Located within this region are the median preoptic nucleus (MnPO), as well as efferent nerve fibers of the subfornical organ (SFO) and the organum vasculosum of the lamina terminalis (OVLT) (Brody et al., 1978; Brody & Johnson, 1980). Over the years, our laboratory and others have dedicated extensive research efforts to dissecting the distinct functions of these components in the context of the hypertensive response to circulating hormones like angiotensin II (AngII) and DOCA.

Both the SFO and the OVLT are circumventricular organs (CVOs), which are unique in that they lack the typical blood–brain barrier, making them directly accessible and responsive to circulating substances, including hormones (Johnson & Gross, 1993). Consequently, extensive research has focused on their roles in maintaining homeostasis in response to changes in osmolarity and the influence of circulating hormones like AngII and aldosterone (Johnson & Gross, 1993).

Moreover, both of these CVOs project to the MnPO (Johnson & Gross, 1993), a critical hypothalamic relay center that sends projections to the paraventricular nucleus (PVN) (McKinley et al., 1992; Miselis, 1981; Xu & Herbert, 1995), which in turn, projects to sympathetic premotor neurons located in the rostral ventral lateral medulla and to preganglionic sympathetic cell bodies found in the spinal cord (Bains & Ferguson, 1995; Stocker & Toney, 2005). In our laboratory, we have investigated all three of these components of the AV3V region and their individual roles in mediating the effects of chronic AngII hypertension. Our findings have revealed that targeted lesions in each of these areas (SFO, OVLT, and MnPO) result in a significant attenuation or near abolition of AngII‐induced hypertension (Collister & Hendel, 2005; Hendel & Collister, 2005; Ployngam & Collister, 2007; Vieira et al., 2010).

On the contrary, our research findings have shown that while the OVLT does indeed play a role in mediating the effects of DOCA salt‐induced hypertension (Collister et al., 2018), the SFO does not significantly contribute to the development of DOCA‐salt hypertension (Osborn et al., 2006). As previously noted, lesions encompassing the entire anteroventral portion lining the third ventricle (AV3V) region have been demonstrated to mitigate DOCA‐salt hypertension (Songu‐Mize et al., 1982). Given the OVLT projects to the median preoptic nucleus (MnPO), we aimed to further elucidate this pathway in the current study. We hypothesized that an intact MnPO is essential for both the development and maintenance of DOCA‐salt hypertension. To test this hypothesis, we conducted telemetric recordings of blood pressure in rats subjected to chronic DOCA‐salt treatment, and compared those with MnPO lesions to sham‐operated rats.

## 2 METHODS

All experimental protocols and procedures received approval from the University of Minnesota Institutional Animal Care and Use Committee (IACUC) and were carried out in accordance with the guidelines set by the National Institutes of Health. Adult male Sprague‐Dawley rats (Charles River Laboratory, Wilmington, MA, USA) weighing between 275 and 300 g were employed in all experiments. The animals were accommodated in a facility approved and monitored by the IACUC, following a 12‐h day/night light cycle with lights on at 7:00 am.

### 2.1 Surgical procedures

Rats were randomly allocated to either the MnPO‐lesioned (MnPOx; *n* = 7) or sham‐lesioned (MnPOsham; *n* = 5) group following previously established procedures (Ployngam & Collister, 2007). In brief, rats were anesthetized with an intraperitoneal injection of ketamine (75 mg/kg) and xylazine (10 mg/kg). Once in a state of surgical anesthesia, the rats were positioned in a stereotaxic apparatus with the head level and secured. A dorsal midline incision was made in the skin of the skull, exposing Bregma and Lambda, which were realigned to be on the same horizontal plane. A 2.0 mm hole was drilled into the skull. A 0.2 mm (0.008 inch) diameter Teflon‐coated monopolar tungsten electrode, with 0.5 mm exposed at the tip, was carefully inserted into the brain at the following coordinates: (−0.25, 7.4), (−0.25, −7.6), (−0.4, −6.1), (−0.4, −7.2) mm from Bregma on the midline, and mm below the dura mater, respectively. Electrolytic lesions were conducted at each location using a cathode current (1.0 mA for 5 s). For sham rats, the same surgical procedures were followed, except ventral coordinates were adjusted by 4.0 mm less to avoid damaging the MnPO, and no current was applied. The skull hole was mended with bone wax, and the skin was closed using 3–0 silk suture. Post‐surgery, rats received an injection of the antibiotic gentamicin (2.5 mg, IM) and the analgesic buprenorphine (0.02 mg, SC).

**FIGURE 1:**
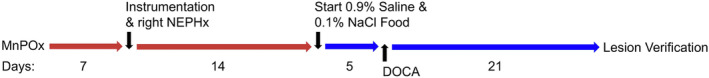
Timeline of study protocol and surgical procedures. MnPOx, electrolytic ablation of the median preoptic nucleus; NEPHx, nephrectomy; DOCA, deoxycorticosterone acetate. 24 h measurements of mean arterial pressure, heart rate, food intake, water intake, and urine output were taken beginning 5 days prior to the start of DOCA treatment and continued through the remainder of the protocol.

One week following the lesion or sham operation (refer to Figure 1), rats underwent a right unilateral nephrectomy and were equipped with radiotelemetric pressure transducers (model no. TA11PAC40; Data Sciences International, Inc., Saint Paul, MN, USA) for continuous 24‐h monitoring of mean arterial pressure (MAP) and heart rate (HR), as detailed previously (Collister et al., 2018; Hendel & Collister, 2005; Ployngam & Collister, 2007; Vieira et al., 2010). Anesthesia was induced using 2% isoflurane. The right ureter and renal vessels were isolated, ligated and cut, leading to the removal of the kidney. Subsequently, the descending aorta was exposed and clamped proximally. A small puncture hole was made in the distal aorta, and the catheter was implanted directly into the aorta via a 21‐guage needle. The catheter was advanced cranially so the tip was located just distal to the renal arteries. The device was secured to the internal abdominal wall using 3–0 silk, and the incision was closed with surgical staples. Rats were allowed to recover on a heating pad and received antibiotics and analgesics, as previously described.

### 2.2 Experimental protocol

Following a 2‐week recovery period, all rats were individually housed in metabolic cages (Nalg Nunc, Rochester, NY) and placed on a diet of 0.1% NaCl (Research Diets, New Brunswick, NJ), with ad libitum access to a 0.9% NaCl drinking solution. After a period of 5 days for baseline measurements, rats were briefly anesthetized with isoflurane, and a 100 mg pellet of DOCA (Sigma Aldrich, catalog number: D7000‐5G) was subcutaneously implanted in each rat. Daily assessments were conducted, including measurements of blood pressure, heart rate, saline and food intake, as well as urine output, for 5 days during the control period and 21 days following the DOCA implantation (refer to Figure 1).

Daily food and water intake, as well as urine output, were determined gravimetrically. Sodium intake was computed as the sum of sodium derived from saline intake (0.154 mEq/mL) and the product of food intake and the sodium content of the food (0.1% NaCl, 0.0175 mmol/g). Urinary sodium concentration was measured using an ion‐specific electrode (Beckman‐Coulter AU680, Beckman‐Coulter, Inc., Brea, CA). The daily urinary sodium excretion was calculated as the product of urine output and urinary sodium concentration. Daily sodium and water balances were determined as the difference between intake and urinary excretion of sodium and water, respectively.

At the conclusion of the experiment, rats were euthanized by administering a lethal dose of pentobarbital sodium. Subsequently, they were perfused transcardially with a 4% paraformaldehyde solution in PBS. Brains were then collected, frozen, and cut coronally into 50 μm sections. These sections were stained with cresyl violet (Sigma Aldrich, catalog number: C0775‐25G) and examined using light microscopy to verify the precise location of the MnPO lesion.

### 2.3 Statistical analysis

The results are presented as means ± standard error (SE). Statistical comparisons were conducted using two‐way analysis of variance (ANOVA) in conjunction with a Student–Newman–Keuls test for post hoc analyses. Statistical significance was considered at *p* < 0.05.

## 3 RESULTS

**FIGURE 2:**
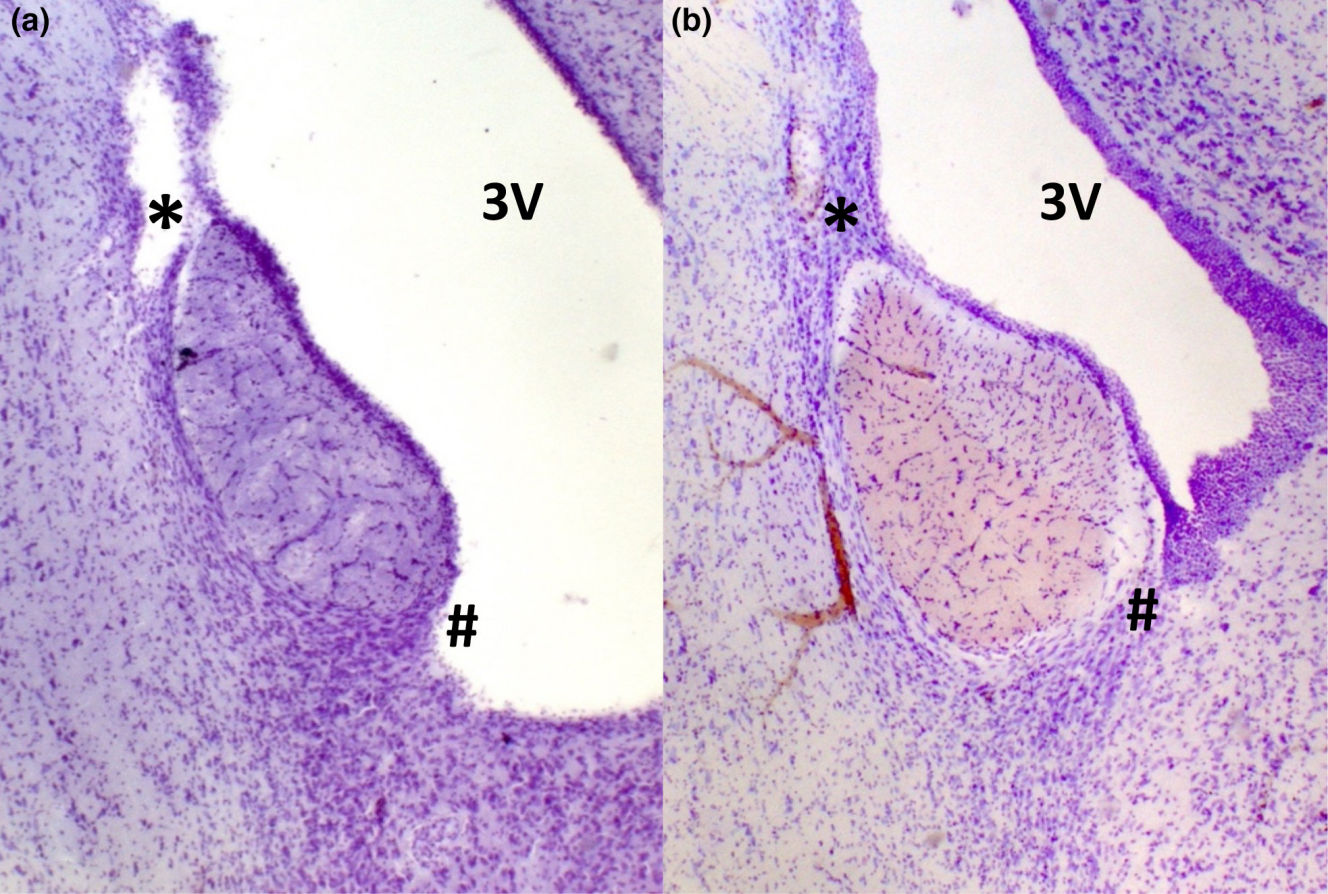
Photomicrographs of 50 μm mid‐sagittal sections of the Median preoptic nucleus (MnPO). (a) Section from a MnPOx rat demonstrating ablated dorsal and ventral MnPO (* and #, respectively). (b) Section from a sham‐lesion rat demonstrating dorsal and ventral MnPO (* and #, respectively). 3 V = Third ventricle.

Figure 2 illustrates representative sagittal sections from a MnPO‐lesioned rat (left panel A) and sham‐lesioned rat (right panel B).

### 3.1 Effect of MnPO lesion on DOCA‐hypertension

**FIGURE 3:**
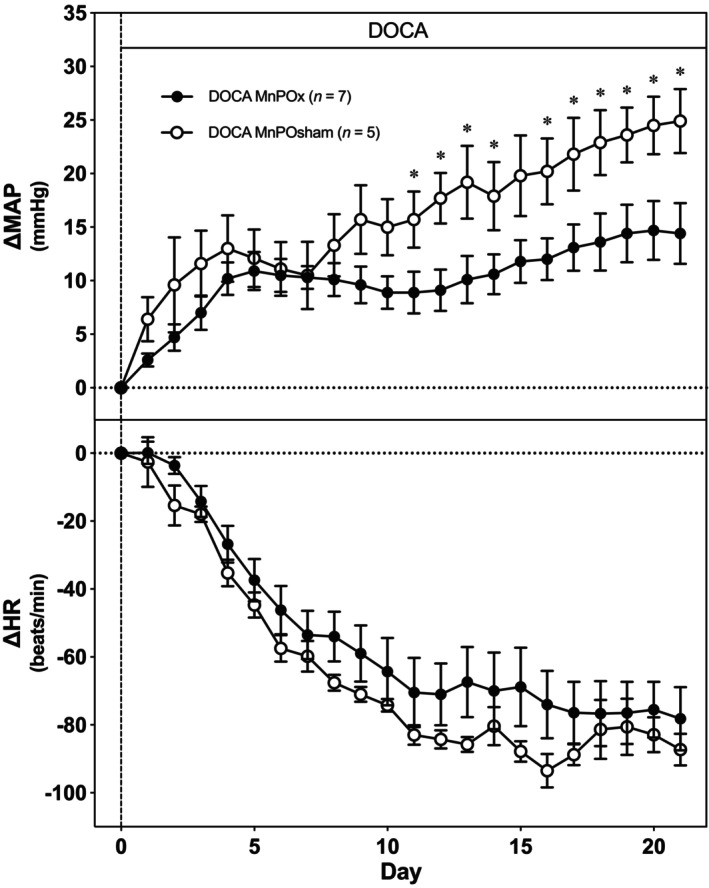
Average change from baseline in 24‐h mean arterial pressure (top) and heart rate (bottom) recorded for 21 days after DOCA implantation (100 mg pellet SQ/rat) in MnPO and sham‐lesioned rats. *p < 0.05 between lesioned and sham rats.

The cardiovascular responses to 21 days of DOCA in MnPO‐lesioned and sham‐lesioned rats are depicted in Figure 3. Baseline MAP did not exhibit differences between groups during the 5‐day average control period (MnPOx; 104 ± 1 mmHg and MnPOsham: 107 ± 2 mmHg). Both groups of rats demonstrated an increase in MAP in response to DOCA; however, the chronic pressor response in MnPO‐lesioned (MnPOx) rats was attenuated by Day 11 of treatment and persisted, with MAP increasing by 25 ± 3 mmHg in MnPOsham rats by Day 21 of DOCA, compared to only a 14 ± 3 mmHg increase in MnPOx rats (Figure 3, top panel). HR did not differ between MnPO‐lesioned and sham groups during the 5‐day control period (MnPOx; 433 ± 6 beats/min and MnPOsham: 437 ± 9 beats/min). Both groups exhibited similar decreases in HR throughout the 21 days of DOCA treatment (Figure 3, bottom panel).

### 3.2 Effect of MnPO lesion on sodium and water balance

**FIGURE 4:**
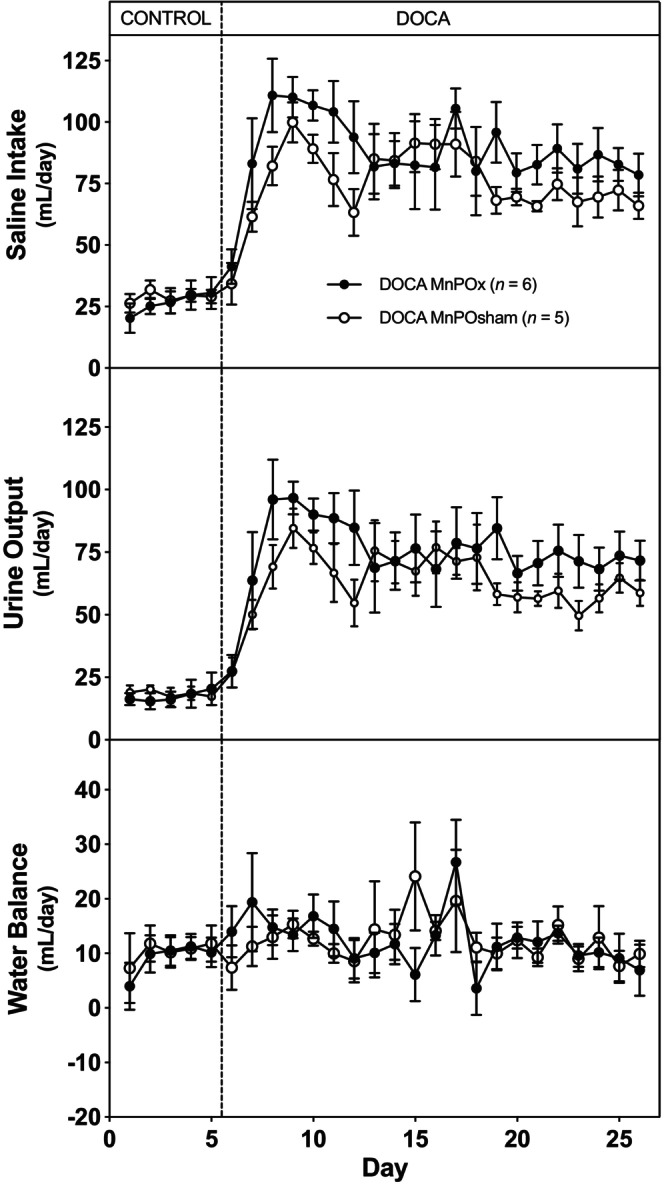
Average 24‐h saline intake (top), urine output (middle), and water balance (bottom) during 5 days of control period (rats began drinking saline) followed by 21 days after DOCA implantation (100 mg pellet SQ/rat) in MnPO and sham‐lesioned rats.

In Figure 4, the data illustrate saline intake, urine output, and water balance. Despite an increase in saline intake induced by DOCA in both groups of rats, this was counteracted by a corresponding rise in urinary output, resulting in no statistically significant difference in overall water balance between the lesioned and sham‐operated rats.

**FIGURE 5:**
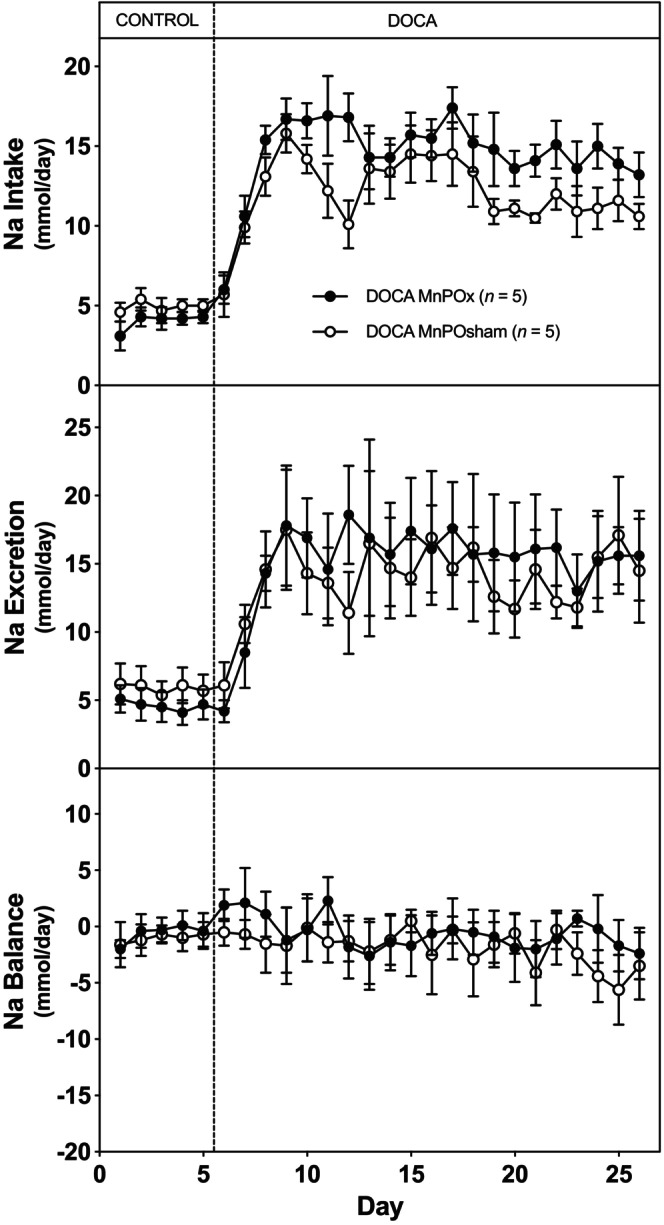
Average 24‐h sodium intake (top), sodium output (middle), and sodium balance (bottom) during 5 days of control period (rats began drinking saline) followed by 21 days after DOCA implantation (100 mg pellet SQ/rat) in MnPO and sham‐lesioned rats.

Figure 5 depicts the sodium intake, sodium excretion, and sodium balance. Similar to water balance, there was no statistically significant difference in sodium balance between the two groups throughout the entire experimental protocol.

## 4 DISCUSSION

In the current study, we aimed to test the hypothesis that the MnPO is necessary for the full development of DOCA‐salt hypertension in the rat. Our results demonstrated that an intact MnPO is important for this chronic hypertensive response and critical for its full development, as rats with lesions of the MnPO had marked attenuation of the hypertensive effects of DOCA‐salt that manifested itself on day 11 of treatment and lasted through the end of the protocol (day 21).

These results align with historical evidence of a central neurogenic component and sympathetic nervous system involvement in the DOCA‐salt model. As such, lesions of the entire AV3V region of the hypothalamus have been shown to eliminate DOCA‐salt hypertension (Buggy et al., 1977; Songu‐Mize et al., 1982), and sympathetic nervous system activity has been shown to be elevated during DOCA‐salt hypertension as well (De Champlain & Eid, 1989; Takeda & Bunag, 1980). Because the AV3V encompasses such a large area including the OVLT, efferent fibers of the SFO, and MnPO, we previously sought to better understand the role of individual components of this critical forebrain area. To that end, our laboratory has shown that discrete lesions of the OVLT, an osmosensitive CVO, markedly reduced DOCA‐salt hypertension (Collister et al., 2018). In order to further dissect the central structures involved in this potential sympathoexcitatory pathway, we investigated the role of the downstream MnPO in the response to DOCA. Consistent with this notion are the known connections of the MnPO to sympathetic regulatory centers. Neurons of the OVLT project to the MnPO, which provides input to downstream neurons of the paraventricular nucleus (PVN), which in turn project to the rostral ventral lateral medulla (RVLM) (Adams et al., 2009). Therefore, our current results regarding this network support the idea of DOCA‐salt acting at the OVLT and driving the hypertensive response via the MnPO.

The intriguing aspect of our findings lies in the striking resemblance of our current data to previous studies, which taken together suggest a common pathway from the brain to the peripheral sympathetic nervous system mediating a large component of the hypertensive response to DOCA‐salt. For example, Abrams, et al. demonstrated an attenuated rise in blood pressure to chronic DOCA in rats treated ICV with the epithelial sodium channel blocker, benzamil (Abrams et al., 2010). In this study, arterial pressure in both control and treated rats responded similarly during the initial 4–5 days of treatment but then diverged, and benzamil treated rats showed a diminished rise in blood pressure during a development phase of 15 subsequent days (Abrams et al., 2010). They described this as the initiation phase which was accompanied by a profound fluid increase for several days in both groups of rats. Our present and previous data from OVLT‐lesioned rats (Collister et al., 2018) mirror these results in both the pattern and magnitude of the pressure response to DOCA‐salt, as well as initial increases in sodium and water, suggesting that the development phase of DOCA‐salt hypertension is centrally mediated, integrating CVO signaling in the OVLT, as well as subsequent downstream neuronal inputs to the MnPO.

With regard to studies involving the peripheral sympathetic nervous system, again our forebrain lesion studies in the DOCA‐salt model are noticeably similar to prior renal denervation and celiac ganglionectomy studies in this model. After an initial similar rise in arterial pressure for 4–5 days, Jacob et al. reported by Day 20 of DOCA that arterial pressure had risen only 13 ± 2 mmHg in renal denervated rats compared to 23 ± 5 mmHg in sham‐operated rats (Jacob et al., 2005). As noted above, in the present study at Day 21, we reported a rise of only 14 ± 3 mmHg in MnPO‐lesioned rats compared to an increase of 25 ± 3 mmHg in MnPO sham‐operated rats. More recently, Banek et al. reported that rats with specific afferent renal denervation exhibited the same fully attenuated hypertensive response to DOCA as rats with total renal denervation (Banek et al., 2016). While the hypertension was lessened to the same extent in afferent renal denervated rats as total renal denervated rats, it was noted that the elevated inflammatory components that accompanied the hypertension were only reduced in the total denervated group, suggesting a potential link between the OVLT‐MnPO neural pathway and efferent renal nerve components of hypertension with the rise in inflammation at the kidney, theoretically driving afferent renal nerve activity and ultimately resulting in hypertension (Banek et al., 2016). In addition to renal nerves, sympathetic splanchnic nerves have been shown to play a role in DOCA‐salt hypertension. Kandlikar and Fink reported similar findings in that by Day 28 of DOCA‐salt, mean arterial pressure had increased only by 15.6 ± 2.2 mmHg in celiac ganglionectomized rats compared to 25.6 ± 2.2 mmHg in sham rats (Kandlikar & Fink, 2011). Along with the renal denervation studies, these findings also mimic almost identically with the results of both our previous study of OVLT‐lesioned rats and the present study using rats with lesions of the MnPO. Taken together, these collective results suggest the existence of a potential final common mediating pathway from the brain (OVLT‐MnPO) to the peripheral nervous system.

In summary, our current findings highlight the critical role of the MnPO in mediating chronic hypertensive effects of DOCA‐salt in the rat. These data, in combination with our results in OVLT‐lesioned rats, strongly suggest a dominant role of the central OVLT‐MnPO axis in mediating the hypertension in this model. Nevertheless, in all our lesion studies, we have never fully blocked the rise in arterial pressure from chronic DOCA‐salt. One possibility is that the delayed onset phase of DOCA‐salt hypertension is also mediated, in part, by non‐neurogenic mechanisms (i.e., salt and water retention). We do not believe this to be the case as we did not observe any changes in chronic water and or sodium balance in our studies. Additionally, while we have previously reported no effect of SFO lesion in this model, we cannot rule out the possibility of CVO redundant pathways in this regard as others have reported. For example, drinking responses to ICV hypertonic saline were only slightly affected by single lesions of the OVLT, SFO, or MnPO (McKinley et al., 1999). Only after all three sites were lesioned was the effect abolished (McKinley et al., 1999). Furthermore, with regard to the peripheral sympathetic nervous system, it could be possible that either splanchnic or renal nerves play a dominant role in this response and by denervation of one or the other, there is a compensatory response in the peripheral sympathetic drive to the other region maintaining a certain level of hypertensive stimulus. Additional dual lesion or denervation studies are needed to rule out these possibilities of redundant compensatory mechanisms in this hypertensive model.

## 5 LIMITATIONS

While not ideal, we acknowledge that these studies were conducted only in male rats. While the justification is that we have numerous studies from our laboratory and others that we can compare the results in chronic hypertensive rat models where we have examined the roles of circumventricular organs, this is not acceptable moving forward and we are currently, and in the future examining the sex differences in this model and others, and the roles of circumventricular organs in the pathogenesis of hormonally induced hypertension.

## FUNDING INFORMATION

This study was supported by University of Minnesota Grant‐In‐Aid of Research, Artistry and Scholarship #546839 and a University of Minnesota Lillehei Heart Institute Collaborative Pilot Grant (UCMED09690).

## CONFLICT OF INTEREST STATEMENT

None.

## Data Availability

The original data used to generate the current results are stored at the University of Minnesota and available upon request of the corresponding author.
